# *MYC* DNA Methylation in Prostate Tumor Tissue is Associated with Gleason Score

**DOI:** 10.3390/genes12010012

**Published:** 2020-12-24

**Authors:** Kathryn Hughes Barry, Kareshma Mohanty, Patricia A. Erickson, Difei Wang, Jianxin Shi, Gary Rose, Ashley Cellini, Kimberly Clark, Nicholas Ambulos, Jing Yin, Liying Yan, Matthew Poulin, Ann Meyer, Yuji Zhang, Søren M. Bentzen, Allen Burke, Arif Hussain, Sonja I. Berndt

**Affiliations:** 1 Department of Epidemiology and Public Health, University of Maryland School of Medicine, Baltimore, MD 21201, USA; kmohanty@som.umaryland.edu (K.M.); patricia.erickson@som.umaryland.edu (P.A.E.); yuzhang@som.umaryland.edu (Y.Z.); sbentzen@som.umaryland.edu (S.M.B.); 2 Program in Oncology, University of Maryland Greenebaum Comprehensive Cancer Center, Baltimore, MD 21201, USA; nambulos@som.umaryland.edu (N.A., Jr.); jyin@som.umaryland.edu (J.Y.); ahussain@som.umaryland.edu (A.H.); 3 Biostatistics Branch, Division of Cancer Epidemiology and Genetics, National Cancer Institute, Rockville, MD 20850, USA; difei.wang@nih.gov (D.W.); jianxin.shi@nih.gov (J.S.); 4 Department of Pathology, University of Maryland School of Medicine, Baltimore, MD 21201, USA; garysamuelrose@gmail.com (G.R.); aburke@umm.edu (A.B.); 5 Pathology Biorepository Shared Service, University of Maryland Greenebaum Comprehensive Cancer Center, Baltimore, MD 21201, USA; acellini@som.umaryland.edu (A.C.); kimberly.clark@som.umaryland.edu (K.C.); 6 Department of Microbiology and Immunology, University of Maryland School of Medicine, Baltimore, MD 21201, USA; 7 EpigenDx, Inc., Hopkinton, MA 01748, USA; lyan@epigendx.com (L.Y.); mpoulin@epigendx.com (M.P.); ameyer@epigendx.com (A.M.); 8 Department of Medicine, University of Maryland School of Medicine, Baltimore, MD 21201, USA; 9 Baltimore Veterans Administration Medical Center, Baltimore, MD 21201, USA; 10 Occupational and Environmental Epidemiology Branch, Division of Cancer Epidemiology and Genetics, National Cancer Institute, Rockville, MD 20850, USA; berndts@mail.nih.gov

**Keywords:** aggressive disease, DNA methylation, Gleason score (GS), *MYC*, non-coding RNAs (ncRNAs), prostate cancer, RNA expression, tumor tissue biomarkers

## Abstract

Increasing evidence suggests a role of epigenetic mechanisms at chromosome 8q24, an important cancer genetic susceptibility region, in prostate cancer. We investigated whether *MYC* DNA methylation at 8q24 (six CpG sites from exon 3 to the 3′ UTR) in prostate tumor was associated with tumor aggressiveness (based on Gleason score, GS), and we incorporated RNA expression data to investigate the function. We accessed radical prostatectomy tissue for 50 Caucasian and 50 African American prostate cancer patients at the University of Maryland Medical Center, selecting an equal number of GS 6 and GS 7 cases per group. *MYC* DNA methylation was lower in tumor than paired normal prostate tissue for all six CpG sites (median difference: −14.74 to −0.20 percentage points), and we observed similar results for two nearby sites in The Cancer Genome Atlas (*p* < 0.0001). We observed significantly lower methylation for more aggressive (GS 7) than less aggressive (GS 6) tumors for three exon 3 sites (for CpG 212 (chr8:128753145), GS 6 median = 89.7%; GS 7 median = 85.8%; *p*-value = 9.4 × 10^−4^). *MYC* DNA methylation was not associated with *MYC* expression, but was inversely associated with *PRNCR1* expression after multiple comparison adjustment (*q*-value = 0.04). Findings suggest that prostate tumor *MYC* exon 3 hypomethylation is associated with increased aggressiveness.

## 1. Introduction

Chromosome 8q24 has been established as an important region in the genetic susceptibility to prostate cancer [1,2,3,4,5,6,7,8,9,10,11], particularly among men of African ancestry, where additional susceptibility single nucleotide polymorphisms (SNPs) have been discovered that are monomorphic in other populations [10] and explain a greater proportion of familial risk [12]. The underlying mechanisms of susceptibility due to this region are still poorly understood. The 8q24 locus has been traditionally described as a gene desert, with the nearest prostate cancer susceptibility SNP located about 200 kb upstream of the oncogene *MYC.* However, this terminology is somewhat misleading, since there are several other genes and non-coding RNAs (ncRNAs) located at 8q24. *MYC* encodes a transcription factor that regulates genes involved in cell growth, differentiation and apoptosis. *MYC* is commonly overexpressed in prostate tumor tissue and has long been thought to play a role in prostate cancer, and particularly with regard to prostate cancer progression [13]. Several other genes and ncRNAs at 8q24, such as *POU5F1B* (previously thought to be a pseudogene), *PRNCR1*, *CASC11* and *CCAT2,* have also been shown to be overexpressed in prostate cancer [14,15,16,17] and may also play a role in the development or progression of prostate cancer.

There is growing evidence for a role of epigenetic mechanisms at chromosome 8q24 in prostate cancer based on the identification of gene regulatory elements at this locus [18], as well as long-range tissue-specific interactions between 8q24 cancer susceptibility loci and *MYC* [19,20]. Expanding on these findings, Du and colleagues examined physical interactions across the genome for several 8q24 cancer susceptibility regions using a chromosome conformation capture (3C)-based, multi-target sequencing technology in cell lines for a variety of cancers (including prostate) and found frequent interactions with *MYC* as well as other intra- and inter-chromosomal targets; other common intra-chromosomal targets included *PVT1*, *FAM84B* and *GSDMC* [21]. The authors also observed an enrichment of interactions with genes in important cancer pathways such as Wnt signaling. Another study investigating genome-wide physical interactions using circularized chromosome conformation capture (4C) coupled with next-generation sequencing for an 8q24 enhancer region also identified interactions with *MYC*, *FAM84B* and *GSDMC*, among other genes [22]. These findings suggest that the 8q24 locus may function as a regulatory hub for a variety of gene targets in important cancer pathways [21].

A major component of the epigenetic code is DNA methylation, which involves the addition of a methyl group (typically to a cytosine base located 5′ to a guanine, i.e., CpG site) and is thought to influence carcinogenesis by affecting gene expression or genetic stability [23]. Previous studies have demonstrated the importance of DNA methylation alterations in prostate cancer, both early in prostate cancer development and also with regard to prostate cancer progression [24,25,26,27]. In previous work from our group, we reported an increased risk of aggressive prostate cancer associated with higher peripheral blood DNA methylation in *MYC* exon 3 in a large prospective study using pre-diagnostic blood samples, which persisted after adjustment for established 8q24 prostate cancer susceptibility loci [28].

In the present study, our primary aim was to investigate whether *MYC* exon 3 DNA methylation in prostate tumor tissue was associated with tumor aggressiveness based on Gleason score (GS), an intermediate prognostic marker for prostate cancer [29]. Exon 3 in *MYC* is highly conserved across species and, interestingly, hypomethylation at *MYC* exon 3 was observed in human myeloma cell lines compared to normal lymphocytes [30] and correlated with progression from normal tissue to metastatic disease in colorectal tissue [31]. As a secondary aim, we sought to explore the potential downstream consequences of prostate tumor *MYC* DNA methylation by evaluating associations with RNA expression for *MYC* and other nearby genes and ncRNAs in prostate tumor tissue. As another secondary aim, we investigated whether the association between *MYC* DNA methylation and GS varied by race (African American vs. Caucasian) given striking disparities in prostate cancer outcomes by race [32] and growing evidence for a role of biological differences in tumor [33,34,35].

Our findings indicated lower *MYC* DNA methylation in prostate tumor compared to paired normal prostate tissue samples for all CpG sites evaluated, suggesting that these may represent somatic alterations in prostate tissue. In addition, we observed significantly lower *MYC* DNA methylation at three exon 3 CpG sites in more aggressive (GS 7) compared to less aggressive (GS 6) tumors, and we observed a similar pattern for another exon 3 CpG site that approached statistical significance. We observed some evidence of a stronger association between *MYC* DNA methylation and GS for African American than Caucasian men for one of the CpG sites that showed a significant overall association with GS. Incorporation of RNA expression data indicated an inverse association between prostate tumor *MYC* DNA methylation and *PRNCR1* expression in tumor tissue. Additional research is needed to replicate these findings and further investigate the potential role of *MYC* DNA methylation in aggressive prostate cancer using experimental laboratory designs.

## 2. Materials and Methods

### 2.1. Study Samples

**Figure 1:**
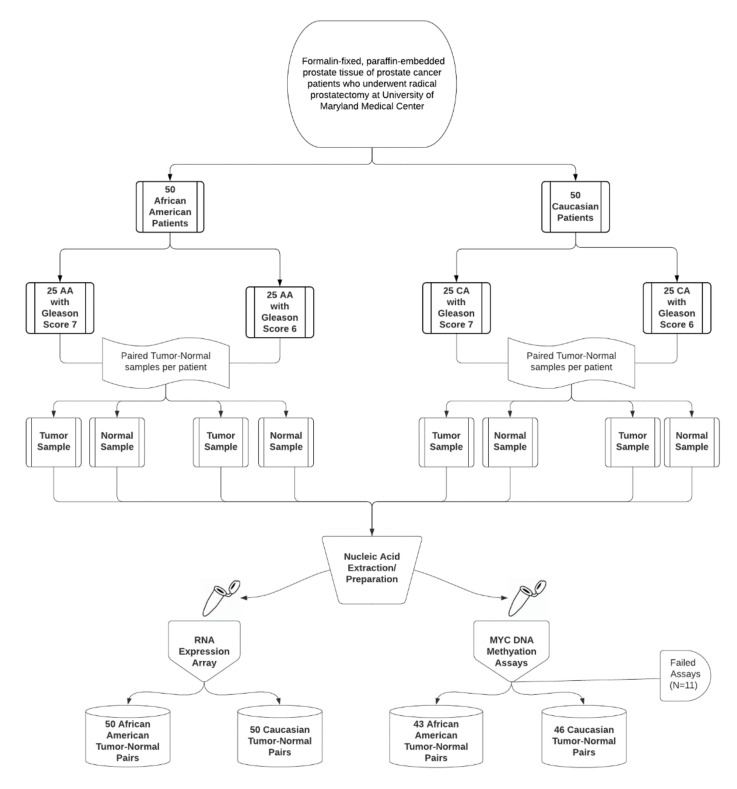
Study workflow for the University of Maryland samples. Abbreviations: AA, African American; CA, Caucasian.

We accessed archival formalin-fixed, paraffin-embedded (FFPE) tumor and normal prostate tissue samples from 50 Caucasian and 50 African American prostate cancer patients who underwent radical prostatectomy at the University of Maryland Medical Center. For each race group, we selected 25 men with GS 7 and 25 men with GS 6 disease to allow for separate evaluation of the association between *MYC* DNA methylation and GS by race; the study workflow is presented in Figure 1. We did not include men with GS 8 or higher due to relatively small numbers available for analysis. We systematically selected the most recent samples available. This study was approved by the IRB at the University of Maryland, Baltimore (HP-00074942, most recent approval date: 7 July 2020). We obtained a waiver of informed consent as part of the approved IRB protocol. The data presented in this paper will be made freely available upon request.

We identified the most representative tumor block and the most representative normal block from the available FFPE radical prostatectomy tissue for each patient. Based on an H&E slide, we circled ~2 mm tumor regions (≥75% purity) and ~2 mm normal regions for nucleic acid extraction, then cut six unstained double-thickness slides (10 µm) per sample, with four used for the DNA methylation assays and two used for the RNA expression assays from the same circled regions. For patients with a GS of 7, we preferentially targeted regions with a Gleason pattern 4 to provide greater contrast with our comparison group (patients with GS of 6, i.e., who typically have Gleason pattern 3 only). We cut additional slides from the tumor and normal samples to serve as replicate samples for quality control for the DNA methylation assays (*n* = 3). We also cut additional tumor and normal slides to serve as quality control replicates for the RNA expression assays (*n* = 5).

### 2.2. Nucleic Acid Extraction/Preparation and RNA Quality Assessment

In preparation for the DNA methylation assays, the University of Maryland FFPE samples were sent to EpigenDx, Inc. (Hopkinton, MA, USA), where direct bisulfite treatment was performed on digested FFPE tissue. Specifically, FFPE samples were deparaffinized with Histochoice clearing reagent and dehydrated with 100% ethanol. Samples were then digested with Proteinase K in a 1X digestion buffer and 20 uL were directly bisulfite treated using the Zymo Research EZ DNA Methylation Direct Bisulfite Kit. For the RNA expression assays, RNA was extracted at the University of Maryland, Baltimore using Qiagen’s RNEasy FFPE kit. Quantitative analysis was performed using both a ThermoFisher NanoDrop and an Agilent Technology’s BioAnalyzer. In addition, we evaluated the quality of the RNA using an RNA quality assessment kit from ThermoFisher. Briefly, this approach involved calculating the difference in quantification cycles (Cq), i.e., delta Cq, between each study sample and high-quality RNA controls (HeLa cells); lower delta Cq values correlate with higher quality RNA (ThermoFisher, Carlsbad, CA, USA).

### 2.3. MYC DNA Methylation Assays

We conducted targeted pyrosequencing assays on bisulfite-treated DNA from the University of Maryland samples to measure DNA methylation at six CpG sites spanning from *MYC* exon 3 to the 3′ UTR (GRCh37/hg19 coordinates: chr8:128753145–chr8:128753221; the sites were labeled as CpG 212–CpG 217 for ease of referencing) (EpigenDx, Inc., Hopkinton, MA, USA). Specifically, five of the CpG sites (212–216) were located in exon 3, and the remaining CpG site (217) was located in the 3′ UTR for *MYC*. The bisulfite conversion step and pyrosequencing assays have been described previously [28,36]. The target sequences for the specific assays run in this study (ADS3573-FS and ADS3573-FS2) are given in Table A1 in Appendix A. There were 11 patients for whom the assays failed in the present study, and thus our analyses using the DNA methylation data were restricted to the remaining 43 African American and 46 Caucasian men (*n* = 89). We computed coefficients of variation (CVs) for each CpG site based on the three participants with replicate QC samples (i.e., three pairs of tumor samples and three pairs of normal samples). The average CV across all six CpG sites was 2.7% for the normal tissue pairs and 3.4% for the tumor tissue pairs.

### 2.4. RNA Expression Data

We obtained RNA expression data from the University of Maryland samples using the Human Clariom D^TM^ array (ThermoFisher, Carlsbad, CA, USA), which covers 138,745 transcript cluster IDs (TCs). We used the UCSC Genome Browser to identify genes and ncRNAs within 1 Mb either upstream or downstream of *MYC* based on GRCh37/hg19 coordinates for *MYC* (i.e., chr8:128748315–128753680). We then used the NetAffx^TM^ tool [37] to identify the TCs that corresponded to *MYC* and the other genes/ncRNAs of interest in our array data. Of the 33 genes/ncRNAs in the region of interest, nine were not covered on the Clariom D array (*SRMP1P1*, *AC020688.1*, *JX003871*, *BC106081*, *DQ515899*, *AC108714.1*, *BC042052*, *HV975509* and *AC103705.1*). Based on the available genes/ncRNAs, we identified a total of 22 TCs for analysis. We performed SST-RMA normalization and log2 transformation of the expression data using TAC v.4.0 software (ThermoFisher, Carlsbad, CA, USA). Analyses of the RNA expression data by tumor/normal status were conducted in 100 tumor–normal pairs. Analyses of RNA expression by GS were done using 100 tumor samples. Analyses of prostate tumor *MYC* DNA methylation in relation to RNA expression in prostate tumor tissue were limited to the 89 tumor samples with DNA methylation data. We computed CVs for each TC of interest based on the five participants with replicate QC samples (i.e., five pairs of tumor samples and five pairs of normal samples). The average CV across the 22 TCs was 6.8% for the normal tissue pairs and 8.3% for the tumor tissue pairs.

### 2.5. The Cancer Genome Atlas (TCGA) DNA Methylation Data

We incorporated DNA methylation array data from the TCGA-PRAD cohort (498 primary prostate tumor samples) to evaluate two additional *MYC* exon 3 CpG sites nearby (<0.2 kb) the CpG sites evaluated in the University of Maryland samples in relation to tumor/normal status and GS among the tumor samples. We downloaded the TCGA-PRAD Illumina Infinium HumanMethylation450 (HM450) BeadArray level 3 data from https://gdac.broadinstitute.org (data version: 2016_10_28) for all 498 PRAD primary tumor and 50 normal samples. We first extracted the β values of these two CpG probes (cg00163372 and cg08526705) for the 50 tumor–normal paired samples (47 Caucasian and three African American men). In addition, we also extracted the data for all tumor samples with GS 6 (*n* = 45) and GS 7 (*n* = 248) for DNA methylation comparisons by GS. We computed the median β values and the interquartile range (IQR) for the tumor and normal tissue samples, and separately by GS for the tumor samples. The β values (ranging from 0 to 1), widely used to measure the methylation level, were converted to percentages to be comparable to the results for the University of Maryland samples.

### 2.6. Statistical Analysis

Analyses were conducted in SAS Studio 3.8 and R Studio (version 3.6.1). We evaluated Spearman correlations in % DNA methylation for *MYC* CpG-CpG pairings, separately for tumor and normal samples. We used Wilcoxon signed-rank tests to assess differential DNA methylation and RNA expression between paired tumor and normal prostate tissue samples from the same individual. We used Wilcoxon rank sum tests to compare DNA methylation and RNA expression between the GS 6 and GS 7 groups using tumor tissue. We used logistic regression models to compute odds ratios (OR) and 95% confidence intervals (95% CI) for the association between decreasing CpG site DNA methylation in prostate tumor tissue (modeled continuously) and GS (7 vs. 6) overall and separately by race (African American and Caucasian). Additional adjustment for age at surgery, year of surgery or imputed immune cell distribution in the tumor tissue from CIBERSORTx software [38] did not appreciably alter the results, and thus these variables were not adjusted in the final models. We evaluated the interaction between each CpG site and race by including CpG site × race as a cross-product term in the model and testing for significance. We also used a similar approach to assess the interaction between each TC and race with regard to GS. We considered an interaction *p*-value < 0.20 as noteworthy (even if not statistically significant). For all analyses, we defined statistical significance as a *p*-value < 0.05.

We performed principal components analysis to assess potential clustering patterns in the RNA expression data by tissue type (tumor/normal), array run, RNA extraction batch, Positive vs. Negative Area Under the Curve (AUC), which is a QC measure computed by the TAC software, RNA quality (based on delta Cq) and covariates including age at surgery and year of surgery. We did not observe much evidence of clustering for most of the factors examined except for array run, Positive vs. Negative AUC and RNA quality (data not presented).

To assess the association between *MYC* DNA methylation and RNA expression in tumor tissue, we ran separate linear regression models for each of the 22 TCs of interest (dependent variable) in relation to each CpG site (independent variable). We computed q-values reflecting the false discovery rate after multiple comparison adjustment (22 models for each CpG site) using the Benjamini and Hochberg method [39]. Given the clustering in the RNA expression data noted above, we assessed the impact of additional adjustment for array run, Positive vs. Negative AUC and RNA quality (as well as age at surgery and year of surgery) in our models; however, these variables did not appreciably alter the results, and thus were not adjusted in the final models. As a sensitivity analysis, we also ran additional linear regression models restricted to the samples of highest quality (Positive vs. Negative AUC > 0.7) to ensure that the results were not unduly influenced by sample quality.

## 3. Results

### 3.1. Study Population Characteristics

Among the 89 prostate cancer patients from the University of Maryland Medical Center who had available *MYC* DNA methylation data for analysis, age at radical prostatectomy ranged from 42–75 years, with a median age of 58 years; the distribution of age at diagnosis was similar to the age at surgery (Table 1). Year of surgery ranged from 2001 to 2017. Our study sample was about evenly divided between Caucasian and African American men (i.e., 52% and 48%, respectively) and between men with GS 6 and those with GS 7 tumors (45% and 55%, respectively) based on our study design. Of the 49 men with GS 7 tumors, a greater proportion had a Gleason pattern of 3 + 4 than 4 + 3 (Table 1). Most men (67%) had a pathologic tumor stage (pT stage) of 2, 25% had a pT stage of 3 and 8% had missing pT stage information. Of the patients with known nodal involvement, 95% were classified as N0 as expected for this radical prostatectomy population. The median pre-operative prostate-specific antigen (PSA) concentration was 6.2 ng/mL (interquartile range (IQR): 4.8, 8.0). The men with GS 6 tumors were generally similar to those with GS 7 tumors with respect to demographic characteristics, except that the GS 6 patients tended to have radical prostatectomy in earlier years (Table 1).

### 3.2. MYC DNA Methylation CpG-CpG Correlations and Prostate Tumor–Normal Differences

DNA methylation levels at the six *MYC* CpG sites evaluated in the University of Maryland samples were moderately correlated in tumor tissue (Spearman rho: 0.27 to 0.66, *p*-value < 0.01 for all CpG site pairings). Correlations were less pronounced in the normal tissue samples (Spearman rho: −0.08 to 0.57), with fewer results achieving statistical significance at the 0.05 level.

In the normal prostate tissue samples from the University of Maryland, % DNA methylation tended to be relatively high for the six CpG sites evaluated, with the exception of CpG 217 (chr8:128753221), which was the one CpG site evaluated in the 3′ UTR of *MYC* and which displayed a median of 66.66% (Table 2). The median % DNA methylation in the normal samples for the other five CpG sites (located in *MYC* exon 3) ranged from 83.39% to 89.72%. When we compared paired tumor and normal prostate tissue samples from the same individual, we observed significantly lower *MYC* DNA methylation for five of the six CpG sites in the tumor than the normal samples (*p*-value < 0.05). The sixth CpG site (CpG 212 at chr8: 128753145) displayed a similar pattern, with a borderline significant *p*-value (*p* = 0.06). The median tumor–normal difference in % DNA methylation for the six CpG sites ranged from −14.74 to −0.20 percentage points (Table 2). Similar results were observed for the two nearby CpG sites evaluated in TCGA, with both sites showing significantly lower methylation in tumor compared to normal samples (median differences of −6.89 and −5.46 percentage points, respectively; Table 2). Median tumor–normal differences in the University of Maryland samples tended to be similar between Caucasian and African American men. The number of African American men with paired tumor and normal tissue in the TCGA dataset was too small (*n* = 3) for meaningful comparison by race. 

### 3.3. MYC DNA Methylation Differences by GS in Prostate Tumor Tissue

**Figure 2:**
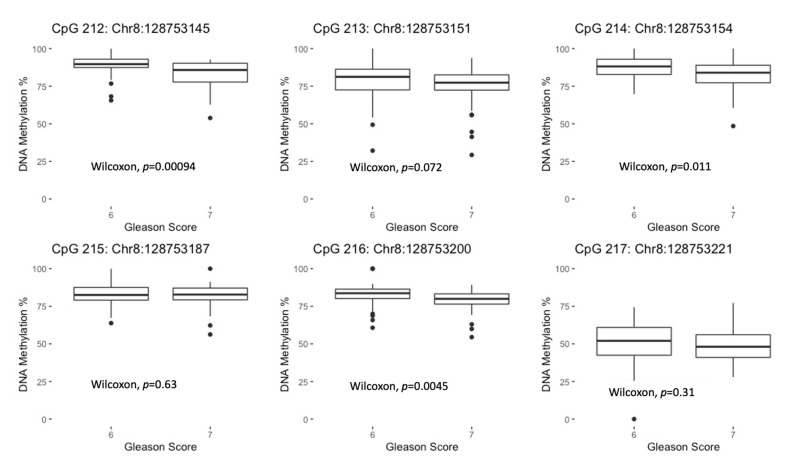
Box plots displaying the median % DNA methylation and interquartile range (25th: 75th percentiles) for *MYC* CpG sites by GS in the University of Maryland prostate tumor samples (*n* = 89).

**Figure A1:**
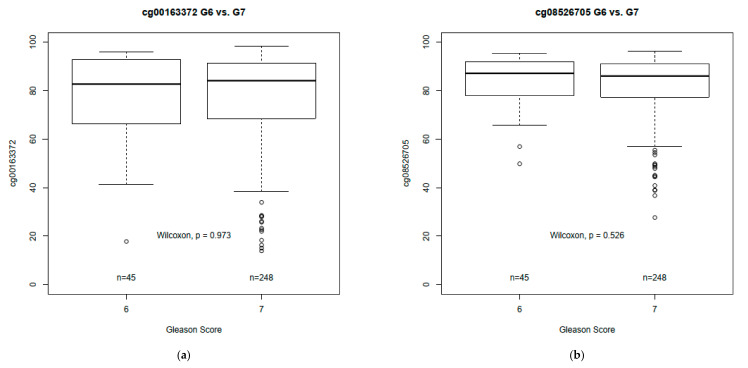
Box plots displaying the median % DNA methylation and interquartile range (25th: 75th percentiles) by Gleason score (GS 6 vs. GS 7) for the two *MYC* exon 3 CpG sites evaluated in The Cancer Genome Atlas (TCGA) data, *n* = 293 prostate tumor samples: (**a**) cg00163372 and (**b**) cg08526705.

Among the University of Maryland tumor samples, we observed significantly lower *MYC* DNA methylation for more aggressive tumors (GS 7) than less aggressive tumors (GS 6) for three exon 3 CpG sites (for CpG 212 (chr8:128753145), median % DNA methylation for GS 6 group = 89.7%; median for GS 7 group = 85.8%; *p*-value = 9.4 × 10^−4^ (Figure 2)). We also observed a similar result for another exon 3 CpG site, CpG 213 (chr8:128753151), which approached statistical significance (*p* = 0.072) (Figure 2). For these four CpG sites, the median difference between the GS groups ranged from 3.7 to 4.1 percentage points. We observed no significant differences by GS for the two CpG sites evaluated in the TCGA dataset (*p* > 0.05 for both) (Figure A1 in Appendix A). Among the CpG sites that displayed a significant overall association with GS, we observed some evidence of a stronger association between lower DNA methylation at CpG 212 (chr8:128753145) and higher GS for African American (OR = 1.23, 95% CI: 1.04–1.45) than Caucasian men (OR = 1.07, 95% CI: 1.00–1.15; *p*-interaction = 0.15; Table 3). 

### 3.4. RNA Expression Tumor-Normal Differences and Differences by GS in Prostate Tumor Tissue

We evaluated differences in the log2-transformed RNA expression levels by tumor/normal sample status for 22 TCs for *MYC* and other genes/ncRNAs within 1Mb of *MYC* using paired tumor–normal prostate tissue samples from the 100 University of Maryland patients (Table 4). Five genes or ncRNAs were significantly upregulated in tumor compared to normal prostate tissue samples, including *MYC*, *PRNCR1*, *RP11-382A18.3*, *POU5F1B* and *RNU4-25P,* whereas one long non-coding RNA (lncRNA), *RP11-255B23.1*, was significantly downregulated in tumor compared to normal prostate tissue (*p* < 0.05). Median tumor–normal differences in expression tended to be similar between Caucasian and African American men (data not presented). Among the 100 tumor tissue samples, only two of the genes/ncRNAs evaluated displayed differential expression between GS 6 and GS 7 samples, which included *CCAT1* and *RNU4-25P* (Table 4). For *CCAT1*, the median log2-transformed expression level (IQR) was 3.5 (3.3, 3.9) for the GS 6 group and 3.2 (3.0, 3.6) for the GS 7 group (*p*-value = 1.5 × 10^−3^); for *RNU4-25P*, the median log2-transformed level (IQR) was 5.8 (5.5, 6.2) for the GS 6 group and 5.3 (4.9, 5.6) for the GS 7 group (*p*-value = 3.6 × 10^−5^; Table 4). Notably, *RNU4-25P* also showed a significant interaction with race with respect to GS (*p*-interaction = 5.6 × 10^−3^), such that there was a significant association between tumor *RNU4-25P* expression and GS among Caucasian men, but not African American men (data not presented). 

### 3.5. Prostate tumor MYC DNA methylation and RNA expression in prostate tumor tissue

When we evaluated prostate tumor *MYC* DNA methylation in relation to RNA expression in prostate tumor tissue in the University of Maryland samples, we did not observe a significant association between any of the six CpG sites and *MYC* expression (data not shown). Our top findings (*p* ≤ 0.05) suggested inverse associations between *MYC* DNA methylation and expression of two lncRNAs at chromosome 8q24, *PRNCR1* and *CASC11*, and a positive association between *MYC* DNA methylation and *miR-1206* expression (Table 5). Notably, the association between DNA methylation at CpG 216 (chr8:128753200) and *PRNCR1* expression remained statistically significant after adjustment for multiple comparisons (β= −0.022, s.e. = 0.007, *p*-value = 2.0 × 10^−3^, *q*-value = 0.04; Table 5). The effect size and direction of the associations remained similar when we restricted our analysis to the 56 prostate tumor samples with the highest quality (Positive vs. Negative AUC > 0.7) (data not shown). The remaining genes and ncRNAs evaluated were not significantly associated with *MYC* DNA methylation (*p* > 0.05).

## 4. Discussion

Based on the importance of the 8q region and the *MYC* gene in prostate carcinogenesis/progression [13,40], we investigated the role of *MYC* DNA methylation (six CpG sites spanning from exon 3 to the 3′ UTR) in prostate tumor tissue from African American and Caucasian prostate cancer patients who underwent radical prostatectomy at the University of Maryland Medical Center. Comparing *MYC* DNA methylation between paired tumor–normal prostate tissue samples from the same men, we found lower DNA methylation in the tumor compared to the paired normal samples for all six CpG sites evaluated, suggesting that these may represent somatic alterations in prostate tissue. Similar patterns were observed for the two nearby CpG sites in exon 3 that were evaluated in TCGA. Interestingly, we also observed lower *MYC* DNA methylation for more aggressive (GS 7) compared to less aggressive (GS 6) tumors for several exon 3 CpG sites. When we incorporated RNA expression data in prostate tumor tissue, we observed significant inverse associations between tumor *MYC* DNA methylation and expression of the lncRNAs *PRNCR1* and *CASC11*, and a significant positive association with *miR-1206* expression. Notably, our finding of an association between CpG 216 (chr8:128753200), one of the CpG sites that displayed a significant association with GS, and *PRNCR1* expression also remained significant after adjustment for multiple comparisons. We did not observe evidence of an association between *MYC* DNA methylation at any of the CpG sites evaluated and *MYC* expression.

A number of epigenome-wide studies have compared DNA methylation at specific CpG sites across the genome between prostate tumor and normal tissue [41,42,43,44,45,46,47,48,49,50,51], with a smaller number of epigenome-wide studies comparing DNA methylation in prostate tissue with respect to GS [52,53,54]. In these studies, *MYC* was not among the top hits, but few, if any, evaluated the specific CpG sites that we evaluated in the present study. For example, most of the previous studies, including TCGA, used array-based technologies such as the Illumina HumanMethylation450 BeadChip that did not cover these specific CpG sites. Another group that used MethylPlex technology with massively parallel sequencing evaluated *MYC* in prostate tumor tissue, but focused on the promoter region and did not evaluate exon 3 [42]. Thus, to our knowledge, our study is one of the first to study these specific *MYC* CpG sites in prostate tissue. Exon 3 in *MYC* is highly conserved across species, and we previously reported a variety of characteristics that are common to transcriptional regulatory regions within 2 kb of the *MYC* exon 3 CpG sites in various prostate cancer cell lines using ENCODE data, which included DNaseI HS peaks, histone methylation and acetylation marks and TFBS for ETV1 and TCF7L2 [28]. Another study using ENCODE data also provided evidence of CHD2 and CTCF transcription factor ChIP-seq clusters nearby *MYC* [55]. CTCF is a multifunctional transcription factor involved in gene expression and gene regulation, and CTCF binding is highly sensitive to DNA methylation [55]. A previous study found lower *MYC* exon 3 DNA methylation in human myeloma cell lines when compared with normal lymphocytes, which correlated with increased expression [30]. Moreover, hypomethylation at *MYC* exon 3 in colorectal tissue was shown to be associated with progression from normal tissue to metastatic disease [31]. Although these latter studies did not focus on prostate cancer, they implicate a potential role of *MYC* exon 3 hypomethylation in the development or progression of cancer, which is consistent with the directions of our findings with regard to prostate tumor–normal tissue differences and differences by GS in prostate tumor tissue.

Although the results should be interpreted cautiously due to relatively small numbers, it is interesting that we observed some evidence of a stronger association for *MYC* DNA methylation at CpG 212 (chr8:128753145) and GS for African American than Caucasian men. Specifically, while the direction of the association was consistent for the two race groups, the association achieved statistical significance in African American men, but was slightly smaller in magnitude and borderline significant in Caucasian men. Investigations of genetic susceptibility loci at 8q24 in the African Ancestry Prostate Cancer GWAS Consortium identified several loci that were specific to African American men, suggesting the potential for different mechanisms at 8q24 contributing to prostate cancer for African American men when compared with men of other ancestries [10]. Our findings, although warranting replication with larger sample sizes, support the need for continued investigation into the potential differing roles of the 8q24 locus in prostate cancer by race/ancestry.

While the present study using prostate tissue among men with prostate cancer and our previous case-control study using pre-diagnostic peripheral blood DNA are not directly comparable due to differences in study design and the timing and type of samples, we note that the direction of association between *MYC* DNA methylation and aggressive prostate cancer observed in the present study was opposite to that reported in our previous study [28]. The differences in our findings may be due, in part, to differences in DNA methylation by tissue type as it is well established that DNA methylation displays distinct patterns by tissue [56], and may also be due in part to changes in DNA methylation over time due to the carcinogenic process [57]. We observed clear differences in DNA methylation between normal and tumor tissue in this study, suggesting that a somatic alteration occurred. We would not expect DNA methylation patterns in pre-diagnostic lymphocyte DNA to reflect somatic DNA methylation alterations in prostate tissue, such as those identified by our present study.

In the present study, we had the advantage of being able to incorporate RNA expression data so we could investigate the potential function of alterations in *MYC* DNA methylation in prostate tissue in the context of RNA expression modulation. Previous research demonstrated the importance of *MYC* promoter methylation and signaling in the regulation of *miR-27a-5p* in prostate cancer [58], providing impetus for further study of *MYC* DNA methylation with regard to other genes/ncRNAs. Our top associations (*p* < 0.05) for *MYC* DNA methylation and RNA expression included inverse associations with *PRNCR1* and *CASC11* and, notably, the association between *MYC* DNA methylation at CpG 216 (chr8:128753200) and *PRNCR1* remained significant after adjustment for multiple comparisons. Prostate cancer-associated non-coding RNA 1 (*PRNCR1)* is a lncRNA that has been proposed to contribute to prostate carcinogenesis by increasing the looping of androgen receptor (AR)-bound enhancers to AR target genes and, in turn, increasing cell proliferation [59]. Previous studies have observed up-regulation of *PRNCR1* expression in prostate cancer cell lines and in the precursor lesion prostatic intraepithelial neoplasia [15]. Consistent with these findings, we also observed higher expression of *PRNCR1* in prostate tumor compared to paired normal tissue samples in our study.Cancer susceptibility candidate 11 (*CASC11*) is another lncRNA that has been shown to be overexpressed in prostate cancer [16], although we did not detect a difference in *CASC11* expression between the tumor and normal samples in our study. Our observations of lower *MYC* DNA methylation in tumor tissue and inverse *MYC* methylation associations with *PRNCR1* and *CASC11* thus appear to be in line with the biology of these ncRNAs (i.e., if *MYC* methylation is lower in tumor than normal tissue and inversely associated with these lncRNAs, then we would expect to see increased expression of these lncRNAs in tumor, as we and others have observed). Our other top finding (*p* < 0.05) regarding *MYC* DNA methylation and RNA expression was a positive association between *MYC* methylation and *miR-1206* expression, although this finding did not persist after multiple comparison adjustment. *MiR-1206* is part of a lncRNA transcript of the *PVT1* gene and, notably, was found to have lower expression in prostate tumor compared to normal tissue in a previous study [60]. The directions of our observed associations (i.e., lower *MYC* methylation in prostate tumor than normal tissue and positive association between *MYC* methylation and *miR-1206* expression) thus also appear to fit with the biology of *miR-1206*.

Our finding of no association between *MYC* DNA methylation and *MYC* expression in prostate tumor suggests that if *MYC* methylation influences the expression of the above ncRNAs, then it is unlikely to be through an effect on *MYC* expression. Instead, it is possible that there is a direct effect of *MYC* methylation on expression of the ncRNAs. There is some plausibility for this hypothesis based on examples of long-range interactions at chromosome 8q24, which influence gene expression, for example, an interaction between rs378854 (a SNP in linkage disequilibrium with rs620861, an established prostate cancer susceptibility SNP) and *PVT1* [61]. While our findings provide some clues about the potential function of alterations in *MYC* methylation, it is also possible that DNA methylation alterations in this region do not directly affect RNA expression, and that these are independent changes that are both correlated with the carcinogenetic process. Further research is needed in *in vitro* or animal models to assess if there is a causal effect of *MYC* DNA methylation alterations on RNA expression at 8q24 and to clarify the underlying mechanisms.

There were several limitations in our study. Our overall sample size was relatively small and we only included radical prostatectomy patients with a GS of 6 or 7. It would have been preferable to compare patients with GS of 8 or higher (instead of GS 7) to those with GS 6 for better contrast (which we hypothesize would have led to larger effect sizes); however, there were relatively few patients with a GS of 8 or higher in our patient population. We expect this may be due, in part, to the fact that we drew our study participants from radical prostatectomy patients and this surgery is predominantly indicated for patients with localized disease, who usually have a lower GS. To help reduce this limitation, we focused on tumor regions with a Gleason pattern 4 where possible for patients with GS 7 to increase the contrast with our comparison group (GS 6, typically involving pattern 3 only). We only evaluated DNA methylation for a small number of CpG sites in *MYC*, for which we had *a priori* rationale; future studies would benefit from a more comprehensive investigation of DNA methylation across the *MYC* genome, as well as replication of the results for the specific CpG sites of interest in an independent population. While inclusion of the TCGA data allowed us to evaluate two CpG sites nearby, we cannot directly compare our results from the University of Maryland samples with TCGA because different CpG sites were evaluated (the sites evaluated in the University of Maryland samples were not covered by the array used in TCGA) and the extent of correlation between the CpG sites is unknown. There were also some ncRNAs that we were unable to evaluate in our analysis because they were not covered on the Clariom D array; however, we were able to evaluate expression for most genes and ncRNAs within 1 Mb of *MYC*, providing insight regarding potential mechanisms. We were also unable to investigate the main effect of race on *MYC* DNA methylation due to our study design, such that we fixed the distribution of GS within each race group, which may have made the distribution of DNA methylation more similar between the race groups as well. However, the rationale for this design is that it maximized our power to separately evaluate the association between *MYC* DNA methylation and GS by race, which was one of our study aims.

Our study also had several strengths, including the integration of epigenetic and transcriptomic data. By incorporating RNA expression data from the Human Clariom D array, we were able to evaluate the association between *MYC* DNA methylation and RNA expression for *MYC* and a variety of other genes and ncRNAs of interest nearby. Other strengths were: (1) our diverse study population, which allowed for separate study of African American men, who bear a disproportionate burden of aggressive prostate cancer and prostate cancer mortality, but have been traditionally understudied; (2) use of GS as an outcome, which is an established intermediate prognostic marker for prostate cancer [29]; (3) centralized pathology review of all samples by a single pathologist; and (4) use of pyrosequencing, a reproducible and quantitative method in detecting inter-individual differences in DNA methylation.

In summary, focusing on CpG sites from exon 3 to the 3′ UTR of *MYC*, our results suggested lower *MYC* DNA methylation in prostate tumor compared to paired normal prostate tissue, and lower exon 3 DNA methylation for more aggressive compared to less aggressive tumors. Although numbers were relatively small, we also noted some evidence of a stronger association for African American than Caucasian men for one of the CpG sites that displayed a significant overall association with GS, highlighting the importance of continued investigation of prostate cancer biomarkers separately by race. Incorporation of RNA expression data allowed for initial investigation into the potential function of these DNA methylation alterations. More research is needed to replicate these findings and further explore the relationship of *MYC* DNA methylation with aggressive prostate cancer, including the function, timing and stability of these alterations over the course of prostate carcinogenesis, and their potential role as passengers or drivers. Further research is also needed to elucidate differences in prostate tumor biology by race/ancestry, which may help provide insight into differences in disease aggressiveness and disparities in prostate cancer outcomes.

## Figures and Tables

**Table 1:** Characteristics of radical prostatectomy prostate cancer patients at the University of Maryland Medical Center who had available *MYC* DNA methylation data, overall and by total GS.

Characteristic	Overall (*n* = 89)	GS 6 (*n* = 40)	GS 7 (*n* = 49)
Age at surgery, median (range)	58 (42, 75)	56.5 (42, 72)	58 (47, 75)
Age at diagnosis, median (range)	57 (42, 75)	56 (42, 72)	57.5 (47, 75)
Surgery year, *n* (col %)			
2001–2009	18 (20.2)	18 (45.0)	0 (0.0)
2010–2017	71 (79.8)	22 (55.0)	49 (100.0)
Race, *n* (col %)			
Caucasian	46 (51.7)	21 (52.5)	25 (51.0)
African American	43 (48.3)	19 (47.5)	24 (49.0)
Gleason pattern, *n* (col %)			
3 + 3	40 (44.9)	40 (100.0)	0 (0.0)
3 + 4	35 (39.3)	0 (0)	35 (71.4)
4 + 3	14 (15.7)	0 (0)	14 (28.6)
pT stage, *n* (col %)			
2	60 (67.4)	29 (72.5)	31 (63.3)
3a	11 (12.4)	2 (5.0)	9 (18.4)
3b	7 (7.9)	1 (2.5)	6 (12.2)
3—not specified	4 (4.5)	1 (2.5)	3 (6.1)
Unknown	7 (7.9)	7 (17.5)	0 (0.0)
pN status, *n* (col %)			
N0	55 (61.8)	15 (37.5)	40 (81.6)
N1	3 (3.4)	0 (0.0)	3 (6.1)
Unknown	31 (34.8)	25 (62.5)	6 (12.2)
PSA (ng/mL), median (IQR)	6.2 (4.8, 8.0)	6.2 (4.7, 7.2)	6.6 (5.0, 9.0)

Abbreviations: GS, Gleason score; IQR, interquartile range; pN status, pathologic nodal involvement; PSA, prostate-specific antigen concentration; pT stage, pathologic tumor stage.

**Table 2:** Tumor–normal prostate tissue differences in % DNA methylation for *MYC* CpG sites in the University of Maryland and TCGA samples.

CpG Site (GRCH37/hg19 Coordinate)	Median % DNA methylation (IQR) for Normal Prostate Tissue Samples	Median % DNA methylation (IQR) for Prostate Tumor Tissue Samples	Median Tumor-Normal Difference in % DNA Methylation (IQR)	Wilcoxon Signed-Rank *p*-Value ^a^
University of Maryland samples (*n* = 89 pairs)				
CpG 212 (Chr8:128753145)	88.27 (85.32, 91.48)	88.29 (82.35, 91.07)	−0.20 (−7.58, 2.91)	0.06
CpG 213 (Chr8:128753151)	85.65 (82.00, 88.48)	78.72 (72.39, 84.20)	−7.36 (−18.40, 0.43)	**1.8 × 10^−8^**
CpG 214 (Chr8:128753154)	89.72 (87.03, 93.23)	86.09 (78.82, 90.38)	−2.98 (−11.38, 2.04)	**4.9 × 10^−5^**
CpG 215 (Chr8:128753187)	86.65 (83.90, 89.65)	82.68 (79.23, 87.12)	−2.84 (−9.79, 0.67)	**1.7 × 10^−6^**
CpG 216 (Chr8:128753200)	83.39 (80.67, 86.29)	81.41 (77.56, 85.22)	−1.88 (−5.70, 2.00)	**3.9 × 10^−3^**
CpG 217 (Chr8:128753221)	66.66 (60.81, 71.15)	49.09 (41.30, 58.63)	−14.74 (−25.53, −6.86)	**9.1 × 10^−19^**
Across all 6 CpG sites	83.23 (81.77, 84.82)	77.40 (73.43, 81.46)	−4.01 (−12.12, 1.32)	**5.9 × 10^−14^**
TCGA samples (*n* = 50 pairs)				
cg00163372 (Chr8:128752987)	94.52 (91.57, 95.50)	84.62 (78.10, 92.01)	−6.89 (−16.92, −2.47)	**9.4 × 10^−^** **^7^**
cg08526705 (Chr8:128753028)	93.01 (91.03, 94.42)	87.30 (80.87, 91.82)	−5.46 (−13.41, −0.32)	**1.3 × 10^−^** **^5^**

Abbreviations: IQR, interquartile range. ^a^ Bolding denotes statistical significance (*p* < 0.05).

**Table 3:** Association between decreasing tumor *MYC* CpG site DNA methylation and tumor aggressiveness (GS 7 vs. 6), overall and by race in the University of Maryland samples ^a^.

Decreasing DNA Methylation (GRCH37/hg19 Coordinate)	Overall, *n* = 89 OR (95% CI)	African American, *n* = 43 OR (95% CI)	Caucasian, *n* = 46 OR (95% CI)	*p*-int ^b^
CpG 212 (Chr8:128753145)	**1.10 (1.03–1.18)**	**1.23 (1.04–1.45)**	1.07 (1.00–1.15)	0.15
CpG 213 (Chr8:128753151)	1.02 (0.99–1.06)	1.06 (0.99–1.14)	1.01 (0.97–1.05)	0.20
CpG 214 (Chr8:128753154)	**1.07 (1.02–1.13)**	1.08 (0.99–1.17)	**1.07 (1.00–1.14)**	0.87
CpG 215 (Chr8:128753187)	1.03 (0.97–1.09)	1.09 (0.98–1.20)	1.00 (0.94–1.07)	0.19
CpG 216 (Chr8:128753200)	**1.08 (1.01–1.16)**	1.15 (0.99–1.32)	1.06 (0.98–1.15)	0.36
CpG 217 (Chr8:128753221)	1.01 (0.98–1.04)	1.01 (0.96–1.06)	1.01 (0.97–1.06)	0.99
Across all 6 sites	**1.01 (1.00–1.02)**	**1.01 (1.00–1.02)**	1.01 (0.99–1.03)	0.89

Abbreviations: CI, confidence interval; GS, Gleason score; OR, odds ratio. ^a^ From logistic regression model (separate model for each CpG site) with decreasing DNA methylation at the CpG site modeled continuously; statistically significant results (*p* < 0.05) are shown in bold. ^b^ *p*-interaction from cross-product term between the CpG site and race in logistic regression model; *p*-interaction values < 0.20 were considered noteworthy.

**Table 4:** List of genes and ncRNAs within 1 Mb of *MYC* and their locations, associated transcript cluster IDs in the Human Clariom D array data and RNA expression differences between paired tumor and normal prostate tissue samples and between GS 6 and GS 7 prostate tumor samples from the University of Maryland.

			Tumor-Normal Difference (*n* = 100 Pairs ^a^)		Differences by GS (*n* = 100 Tumor Tissue Samples ^a^)		
Gene/ncRNA	GRCH37/hg19 Coordinate from UCSC Genome Browser	Human Clariom D TC	Median Difference in log2 Expression (IQR)	Wilcoxon Signed-Rank *p*	GS 6, Median log2 Expression (IQR)	GS 7, Median log2 Expression (IQR)	Wilcoxon Rank Sum *p*
*MYC*	chr8:128748315–128753680	TC0800008845.hg.1	0.2 (−0.2, 0.5)	**0.01**	5.9 (5.6, 6.2)	5.9 (5.7, 6.4)	0.21
**UPSTREAM of *MYC***							
*PCAT1*	chr8:128025399–128033259	TC0800008832.hg.1	0.1 (−0.2, 0.4)	0.06	4.2 (4.0, 4.4)	4.1 (3.9, 4.3)	0.18
*PCAT2*	chr8:128084939–128094466	TC0800012460.hg.1	0.1 (−0.5, 0.7)	0.39	4.0 (3.6, 4.3)	3.9 (3.5, 4.3)	0.69
*PRNCR1*	chr8:128092119–128104840	TC0800008833.hg.1	0.2 (−0.2, 0.7)	**1.6 × 10^−4^**	4.3 (4.1, 4.6)	4.4 (4.1, 4.7)	0.64
*RP11-255B23.1*	chr8:128098508–128099755	TC0800012461.hg.1	−0.7 (−0.3, 0.2)	**0.04**	4.3 (4.1, 4.6)	4.4 (4.2, 4.8)	0.08
*CASC19*	chr8:128200030–128209872	TC0800012462.hg.1	0.0 (−0.2, 0.3)	0.52	3.4 (3.3, 3.7)	3.4 (3.2, 3.7)	0.22
*CCAT1*	chr8:128219627–128231513	TC0800012463.hg.1	0.1 (−0.3, 0.5)	0.10	3.5 (3.3, 3.9)	3.2 (3.0, 3.6)	**1.5 × 10^−3^**
*CASC21*	chr8:128256882–128404876	TC0800008836.hg.1	0.0 (−0.2, 0.4)	0.17	3.7 (3.5, 4.1)	3.7 (3.3, 3.9)	0.35
*RP11-382A18.3*	chr8:128304478–128304685	TC0800008837.hg.1	0.2 (−0.2, 0.8)	**6.8 × 10^−4^**	5.5 (5.3, 5.8)	5.6 (5.1, 6.1)	0.81
*CCAT2*	chr8:128412644–128414395	TC0800008838.hg.1	0.1 (−0.3, 0.3)	0.53	3.4 (3.2, 3.5)	3.3 (3.2, 3.6)	0.83
*POU5F1B*	chr8:128428070–128429455	TC0800008839.hg.1	0.1 (−0.2, 0.5)	**0.02**	5.1 (4.8, 5.4)	5.0 (4.8, 5.2)	0.13
*CASC8*	chr8:128455595–128494384	TC0800012464.hg.1	0.0 (−0.3, 0.5)	0.34	3.2 (3.0, 3.5)	3.3 (3.1, 3.5)	0.70
*LOC727677*	chr8:128455595–128494384	(same as above)					
*CASC11*	chr8:128712853–128746213	TC0800011782.hg.1	0.0 (−0.3, 0.4)	0.40	3.8 (3.4, 4.1)	3.7 (3.4, 3.9)	0.56
**DOWNSTREAM OF *MYC***							
*PVT1*	chr8:128806779–129113499	TC0800008848.hg.1	0.0 (−0.5, 0.5)	0.75	4.6 (4.2, 4.9)	4.4 (4.0, 5.0)	0.72
*miR-1204*	chr8:128808208–128808274	(same as above)					
*TMEM75*	chr8:128958805–128960969	TC0800011789.hg.1	0.0 (−0.3, 0.4)	0.49	3.9 (3.7, 4.1)	3.8 (3.6, 4.1)	0.33
*miR-1205*	chr8:128972879–128972941	TC0800008855.hg.1	0.0 (−0.7, 0.8)	0.92	4.8 (4.2, 5.4)	4.8 (4.5, 5.6)	0.34
*RNU1-106P*	chr8:129011377–129011540	TC0800008856.hg.1	0.0 (−0.4, 0.5)	0.96	13.9 (13.3, 14.5)	14.2 (13.6, 14.9)	0.09
*RNU4-25P*	chr8:129022628–129022857	TC0800011790.hg.1	0.2 (−0.2, 0.5)	**0.02**	5.8 (5.5, 6.2)	5.3 (4.9, 5.6)	**3.6 × 10^−5^**
*RN7SKP226*	chr8:129232750–129232998	TC0800008864.hg.1	−0.1 (−0.7, 0.3)	0.12	5.2 (4.7, 5.7)	5.4 (4.9, 5.9)	0.31
*miR-1206*	chr8:129021144–129021202	TC0800008857.hg.1	0.4 (−0.9, 1.6)	0.07	5.9 (4.1, 7.8)	6.5 (4.9, 8.0)	0.38
*miR-1207*	chr8:129061398–129061484	TC0800008858.hg.1	0.1 (−0.1, 0.3)	0.05	4.2 (4.1, 4.4)	4.2 (4.1, 4.5)	0.86
*miR-1208*	chr8:129162362–129162434	TC0800008862.hg.1	0.0 (−0.2, 0.3)	0.44	4.6 (4.5, 4.9)	4.6 (4.4, 4.8)	0.06

Abbreviations: GS, Gleason score; IQR, interquartile range; ncRNA, non-coding RNA; TC, transcript cluster ID. ^a^ The analyses in this table were based on all 100 prostate cancer patients from the University of Maryland Medical Center since there was no missing RNA expression data for the TCs of interest; statistically significant *p*-values (*p* < 0.05) are shown in bold.

**Table 5:** Top hits for *MYC* DNA methylation and RNA expression in prostate tumor tissue (*p* ≤ 0.05), *n* = 89 tumor tissue samples from the University of Maryland.

Non-coding RNA	Description	Coordinates (GRCh37/hg19)	*MYC* CpG Site and Position (hg19)	Linear regression β (s.e.); *p*-Value (*q*-Value) ^a^
*PRNCR1*	prostate cancer associated non-coding RNA 1	chr8:128092119–128104840	CpG 216 (chr8:128753200) CpG 217 (chr8:128753221)	β = −0.022 (0.007); ***p* = 2.0 × 10^−3^ (*q* = 0.04)** β = −0.0094 (0.0039); ***p* = 0.02** (*q* = 0.20)
*CASC11*	cancer susceptibility candidate 11 (non-protein coding), transcript variant 2	chr8:128712853–128746213	CpG 212 (chr8:128753145) CpG 217 (chr8:128753221)	β = −0.011 (0.005); *p* = 0.05 (*q* = 0.59) β = −0.010 (0.003); ***p* = 4.1 × 10^−3^** (*q* = 0.09)
*miR-1206*	microRNA 1206	chr8:129021144–129021202	CpG 212 (chr8:128753145)	β = 0.053 (0.025); ***p* = 0.03** (*q* = 0.59)

^a^ Bolding denotes statistical significance at the 0.05 level; *q*-values correspond to the false discovery rate after multiple comparison adjustment (22 tests for each CpG site) using the Benjamini and Hochberg approach.

## Appendix A

**Table A1:** *MYC* Pyrosequencing Assay Target Sequences; green highlight denotes the CpG sites covered in the present study.

Assay ID	Genomic Target Sequence	Bisulfite Converted Target Sequence	Pyrosequencing Dispensation Order
ADS3573-FS	ACTTGTTGCGGAAACGACGAGAACAG TTGAAACACAAACTTGAACAGCTACGG AACTCTTGTGCGTAAGGAAAAGTAAGG AAAACGATTCCTTCTAACAGAAATG	ATTTGTTGYGGAAAYGAYGAGAATA GTTGAAATATAAATTTGAATAGTTA YGGAATTTTTGTGYGTAAGGAAAAG TAAGGAAAAYGATTTTTTTTAATAG AAATG	ACTGTAGTCGATCGATCGAGATCAGTGA TCATCACTGATCAGCTGATCGACTCTGT GTCGTCAGAAGTCAGATCGA
ADS3573-FS2	AACTTGAACAGCTACGGAACTCTTGTG CGTAAGGAAAAGTAAGGAAAACGATT CCTTCTAACAGAAATGTCCTGA	AATTTGAATAGTTAYGGAATTTTTGT GYGTAAGGAAAAGTAAGGAAAAYG ATTTTTTTTAATAGAAATGTTTTGA	ACTGACTAGCTGATCGATCTGTAGTCGT AGAAGTAGAATCGA
